# Emphysematous Cystitis Complicated by Pneumorrhachis

**DOI:** 10.7759/cureus.30401

**Published:** 2022-10-17

**Authors:** Jane Ehret, Thomas W Powell, Pang Lam, Valerie Cluzet

**Affiliations:** 1 Internal Medicine, Vassar Brothers Medical Center, Poughkeepsie, USA; 2 Intensive Care Unit, Nuvance Health/Health Quest at Vassar Brothers Medical Center, Poughkeepsie, USA; 3 Infectious Diseases, Vassar Brothers Medical Center, Poughkeepsie, USA

**Keywords:** pneumorrhachis, academic radiology, trauma critical care, critical care and internal medicine education, critical care and hospital medicine, severe sepsis, emphysema cystitis, complicated uti, intensive care medicine, id critical care

## Abstract

Emphysematous cystitis (EC) is a potentially life-threatening urinary tract infection (UTI) characterized by the presence of gas within the bladder wall and lumen. The extension of gas beyond the bladder wall is rare and indicative of severe disease. We present a case of septic shock secondary to EC with the extension of air through the paraspinal and psoas muscles and into the epidural space of the lumbar spinal canal. This finding of intraspinal air is a rare radiographic phenomenon known as pneumorrhachis (PR).

## Introduction

Emphysematous cystitis (EC) is a rare type of complicated urinary tract infection (UTI) caused by an infection with gas-producing bacteria, most commonly *Escherichia coli* or *Klebsiella pneumoniae* [1]. The risk factors for EC include female gender, advanced age, uncontrolled diabetes mellitus, urinary stasis, recurrent UTIs, immunosuppression, neurogenic bladder, and indwelling urinary catheters [2]. Patients with uncontrolled diabetes are at a higher risk due to the presence of glucosuria. The culprit bacteria utilize glucose as a substrate for fermentation, producing carbon dioxide and other gases as a by-product [1]. When compounded with conditions that impair the diffusion of formed gas, there is a disequilibrium between gas formation and clearance. This can be seen in patients with recurrent UTIs or chronic urinary obstruction [2].

The clinical presentation of EC is highly variable, ranging from an asymptomatic incidental finding to septic shock [1,2]. The most common presenting symptoms are nonspecific abdominal pain and hematuria. Urinary complaints are seen in only about 53% of patients [2]. Therefore, EC should be considered in high-risk patients who present with nonspecific symptoms, even in the absence of typical UTI symptoms.

Radiographic evidence of bladder wall emphysema, with or without intraluminal emphysema, is required to diagnose EC. Intraluminal air is less specific as it may occur secondary to bladder instrumentation or diverticular disease or in the presence of anatomical fistulae. In general, EC is less severe than other emphysematous genitourinary infections such as emphysematous pyelonephritis [1,2]. Occasionally, EC may present concurrently with emphysematous pyelonephritis [3,4].

The emphysematous changes seen in EC are usually limited to the bladder wall and lumen. Extension beyond the bladder wall suggests a more serious infection [1]. In rare instances, as seen with our patient, air from the bladder may extend into the surrounding soft tissue and into the epidural space of the spinal canal, a radiographic finding referred to as pneumorrhachis (PR).

## Case presentation

An 88-year-old male with a history of diabetes mellitus, benign prostatic hyperplasia (BPH), recurrent urinary tract infections (UTIs), Crohn’s disease managed with prednisone, and systolic heart failure presented to the hospital with lower back pain for one week. He was found to be encephalopathic with severe urinary retention and septic shock of presumed urinary tract source.

Upon arrival, he was afebrile with a heart rate of 102 beats per minute, blood pressure of 97/64 mmHg, respiratory rate of 22 breaths per minute, and a blood oxygen saturation of 98% on 4 L of supplemental oxygen. On physical examination, he was somnolent and disoriented but responsive to verbal stimuli and neurologically intact. Heart and lung examinations were positive for tachycardia with a regular rhythm, coarse breath sounds bilaterally, and tachypnea. His abdomen was soft and distended with suprapubic tenderness.

Initial serum blood tests revealed a glucose level of 230 mg/dL, leukocytosis with white blood cell (WBC) count of 21.8 × 10^9^/L (93% neutrophils), hemoglobin of 8.3 g/dL, platelet count of 123 × 10^9^/L, acute kidney injury with a blood urea nitrogen (BUN) of 39 mg/dL and creatinine of 3.06 mg/dL, lactic acid level of 6.67 mmol/L, bicarbonate of 18 mEq/L, procalcitonin of 14.2 ng/mL, alkaline phosphatase (ALP) of 304 IU/L, total bilirubin of 2.70 mg/dL, normal aminotransferase levels, and mild hyponatremia of 131 mmol/L (Table 1).

**Table 1:** Initial serum blood tests. WBC: white blood cell; BUN: blood urea nitrogen; ALT: alanine aminotransferase; AST: aspartate aminotransferase; ALP: alkaline phosphatase; g/dL: grams per deciliter; mg/dL: milligrams per deciliter; mmol/L: millimoles per liter; mEq/L: milliequivalents per liter; IU/L: international units per liter; ng/mL: nanograms per milliliter

Serum blood tests	Results
WBC count	21.8 × 10^9^/L (93% neutrophils)
Hemoglobin	8.3 g/dL
Platelet count	123 × 10^9^/L
Glucose	230 mg/dL
BUN	39 mg/dL
Creatinine	3.06 mg/dL
Sodium	131 mmol/L
Potassium	5.3 mmol/L
Chloride	95 mmol/L
Bicarbonate	18 mEq/L
Calcium	8.9 mg/dL
ALT	31 IU/L
AST	31 IU/L
ALP	304 IU/L
Total protein	6.8 g/dL
Albumin	2.7 g/dL
Total bilirubin	2.70 mg/dL
Lactic acid	6.67 mmol/L
Procalcitonin	14.2 ng/mL

Arterial blood gas (ABG) showed mixed metabolic and respiratory acidosis with pH 7.17, partial pressure of carbon dioxide (pCO_2_) of 53 mmHg, partial pressure of oxygen (pO^2^) of 50 mmHg, bicarbonate of 18 mEq/L, and a base deficit of -9.5 mmol/L (Table 2).

**Table 2:** Arterial blood gas (ABG) findings. pH: potential of hydrogen; pCO_2_: partial pressure of carbon dioxide; pO_2_: partial pressure of oxygen; HCO_3-_: bicarbonate; mmHg: millimeters of mercury; mEq/L: milliequivalents per liter; mmol/L: millimoles per liter

Arterial blood gas	Results
pH	7.17
pCO_2_	53 mmHg
pO_2_	50 mmHg
HCO_3-_	18 mEq/L
Base deficit	-9.5 mmol/L

A urinalysis was negative for nitrites but positive for leukocyte esterase, microscopic hematuria, proteinuria, and trace ketones (Table 3).

**Table 3:** Urinalysis findings. WBC: white blood cells; RBC: red blood cells; cfu: colony-forming units; Leu/uL: leukocytes per microliter; pH: potential of hydrogen; mg/dL: milligram per deciliter; HPF: high power field; mL: milliliter *Urine culture results are discussed later.

Urinalysis	Results
Color	Dark brown
Appearance	Turbid
pH	6.0
Specific gravity	1.020
Glucose	Negative
Ketones	Trace mg/dL
Blood	>1 mg/dL
Protein	200 mg/dL
Nitrite	Negative
Leukocyte esterase	500 Leu/uL
WBC	>100/HPF
RBC	>100/HPF
Bacteria	Many/HPF
*Urine culture	>100,000 cfu/mL *Escherichia coli*

Piperacillin-tazobactam was empirically started, and a urinary catheter was placed. The patient rapidly decompensated requiring emergent intubation for respiratory failure, multiple vasopressors for hemodynamic instability, and transfer to the intensive care unit (ICU).

**Figure 1:**
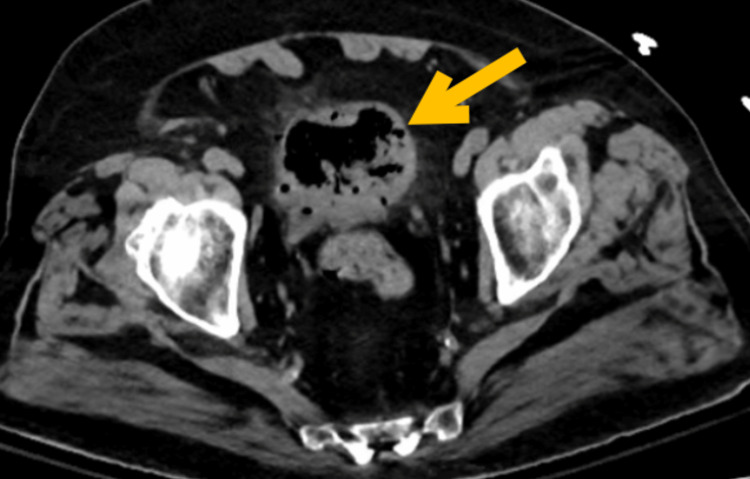
Non-contrast CT of the abdomen and pelvis (axial section through the urinary bladder). Diffuse emphysema throughout the bladder wall with surrounding stranding (arrow). CT: computed tomography

Computed tomography (CT) without contrast of the abdomen and pelvis demonstrated diffuse emphysema throughout the bladder wall consistent with extensive emphysematous cystitis (EC) without hydronephrosis or pyelonephritis (Figure 1).

**Figure 2:**
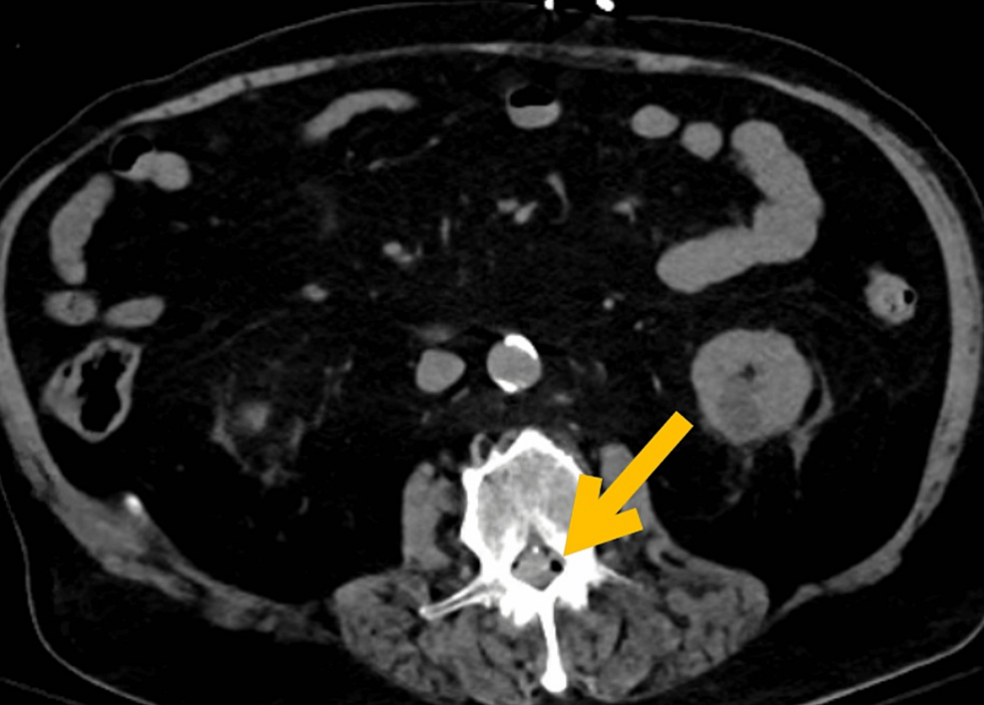
Non-contrast CT of the abdomen and pelvis (axial section through the lumbar spine). Air bubbles within the epidural space of the lumbar spinal canal (arrow). CT: computed tomography

**Figure 3:**
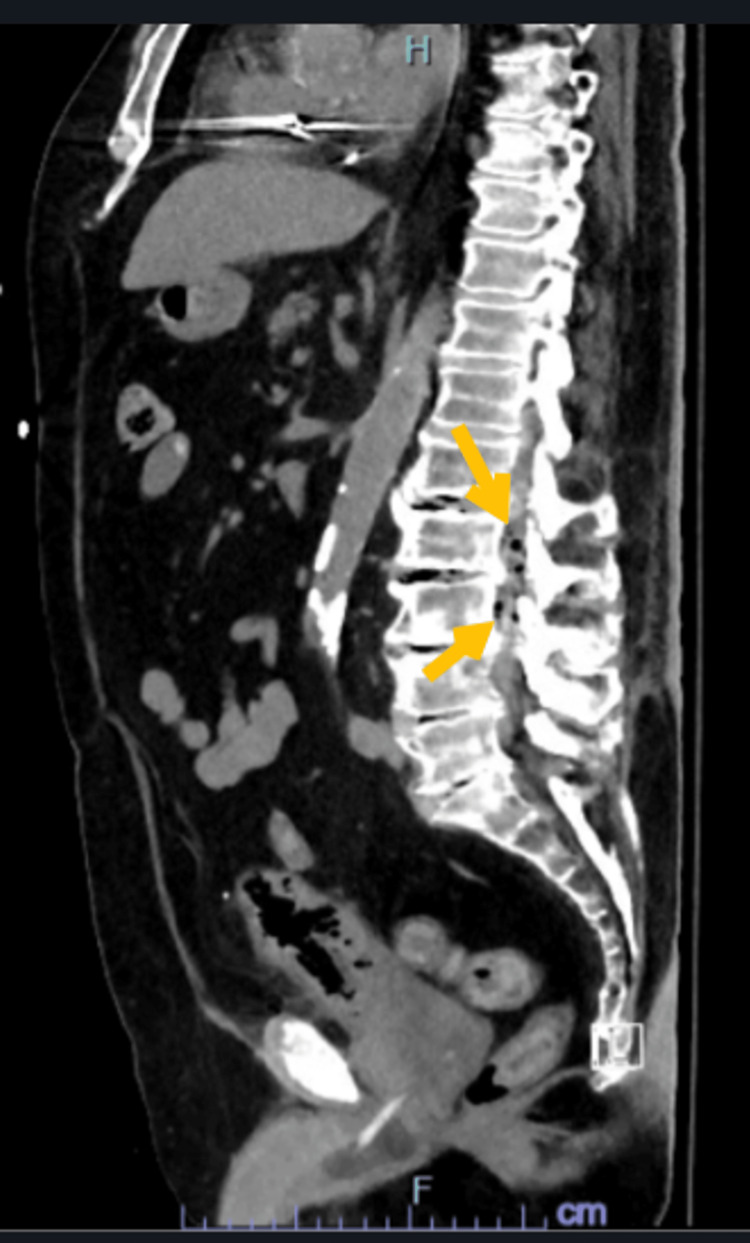
Non-contrast CT of the abdomen and pelvis (sagittal section). Air bubbles extending from the paraspinal muscles into the epidural space of the lumbar spinal canal (arrows). CT: computed tomography

Air bubbles were noted along the right psoas muscle and left paraspinal muscles and within the lumbar spinal canal (Figure 2 and Figure 3).

Urine and blood cultures ultimately grew pan-sensitive *Escherichia coli*, after which the piperacillin-tazobactam was switched to ampicillin-sulbactam. Eventually, he was weaned off vasopressors and extubated. His hospital course was complicated by new-onset atrial fibrillation, aspiration pneumonia, and *Clostridium difficile *colitis. The infection cleared, but the patient never fully recovered. He was discharged to home hospice and passed away of decompensated heart failure a few weeks later.

## Discussion

Pneumorrhachis (PR), the presence of air within the spinal canal, is a radiographic phenomenon that can be caused by a variety of different pathologies. It is almost exceptionally associated with air distributions in other body compartments, such as pneumocephalus, pneumothorax, pneumomediastinum, pneumopericardium, or subcutaneous emphysema [5].

PR can be classified by the location of intraspinal air into extradural PR (air within the epidural space) and intradural PR (air within the subarachnoid space). This anatomical classification is important because these two entities are associated with different pathologies, mechanisms of air entry, and clinical implications [5,6]. Intradural PR usually occurs secondary to major trauma and is often associated with pneumocephalus, basal skull fractures, and tears in the dura mater [7]. When associated with pneumocephalus, patients may present with headaches or focal neurological signs. In some cases, a tear in the dura mater can also serve as a portal for bacteria to enter the subarachnoid space, resulting in meningitis [6]. In contrast, extradural PR is typically innocuous, occurs secondary to a variety of pathologies, and has no clinical manifestations [5,7].

It can be difficult to differentiate between intradural and epidural PR with standard computed tomography (CT). However, the distribution of air can provide a useful clue. In intradural PR, air accumulates in both the anterior and posterior portions of the subarachnoid space. In contrast, dissected air in the epidural space is predominantly posterior to the spinal cord, where loose connective tissue provides a lower resistance compared to the rich vascular network of the anterior epidural space [7].

PR can be classified according to etiology into iatrogenic, traumatic, and nontraumatic PR. Iatrogenic PR is most common and has been seen following lumbar punctures, epidural or spinal anesthesia, and spinal surgeries [8]. Trauma is the second most common etiology and may result in extradural or intradural PR. Traumatic extradural PR has been reported secondary to traumatic pneumomediastinum, traumatic pneumothorax, pelvic or vertebral fractures and herniated discs and with dural-enteric fistula in the setting of gunshot wounds. Traumatic intradural PR is a marker of severe injury and occurs following basilar skull fractures that tear the dura mater or penetrating spinal injuries [7,8].

Nontraumatic PR usually results from an extension of air from a contiguous site, such as pneumomediastinum, spontaneous pneumothorax, pneumoperitoneum, pneumopericardium, or subcutaneous emphysema. A 2021 case-based review of the literature found six cases of PR triggered by upper respiratory tract infections, five in association with asthma exacerbations, three with vomiting caused by diabetic ketoacidosis, three with emphysematous pyelonephritis, and two with degenerative disc disease [9]. Other cases have also been seen with the Valsalva maneuver, foreign body aspiration, inhalational drug use, and malignancy-related fistulae [5,7-9].

Infectious causes of PR are exceptionally rare but have been reported secondary to epidural abscesses, sacral decubitus ulcers, and intra-abdominal or retroperitoneal infections with gas-forming organisms, including emphysematous infections of the genitourinary tract [7,9]. A handful of cases have been reported of PR secondary to emphysematous pyelonephritis [10-13], whereas only one other report of PR secondary EC has been reported [14].

In our patient, we believe that PR was a complication of EC through the dissection of air from the bladder wall, through the surrounding soft tissue, and into the epidural space of the lumbar spinal canal via the neural foramina. The presence of air bubbles within the paraspinal and psoas muscles on the CT scan support this.

The pathophysiology of air entry with nontraumatic PR, including those of infectious origin, involves the extension of air from contiguous sites. The epidural space lacks a protective fascial envelope, allowing for open communication between the retropharyngeal and posterior mediastinum. Because of this anatomical connection, the air within the posterior mediastinum or retropharyngeal space can dissect along the fascial planes into the epidural space. Furthermore, the fascial planes of the retropharyngeal space and posterior mediastinum are continuous with that of the retroperitoneum. This creates a continuum for air travel, such that aberrant air arising in one of these areas can potentially spread elsewhere [15-17].

This anatomical connection explains why nontraumatic PR is usually seen in tandem with pneumomediastinum, pneumoperitoneum, or subcutaneous emphysema [6,7]. For example, respiratory complications that create high intrathoracic pressure cause PR by inducing pneumomediastinum. High airway pressures, such as with violent coughing, induce alveolar rupture, allowing air to spread along the perivascular and peribronchial fascial sheaths and reach the mediastinum, a process known as the Macklin effect [16]. When air accumulates within the peritoneal cavity or retroperitoneum, pneumoperitoneum serves as the driving force for air dissection along the fascial planes into the epidural space [16,17]. Another mechanism is through direct invasion of the pelvic vasculature, after which air migrates into the internal plexus of the spine [7].

By this mechanism, air originating from abdominal or retroperitoneal sources, including those of the urinary tract, can dissect through the paraspinal soft tissues to enter the epidural space through the neural foramina and the neurovascular sheath through the venous plexus [10].

Most cases of PR are self-limiting, as the air reabsorbs spontaneously. Neurosurgical intervention may be warranted in cases of intradural PR secondary to severe injury. Extradural PR is usually managed conservatively [6]. When resulting from an infectious cause, prompt administration of antimicrobials may prevent rare complications of meningitis, discitis, or osteomyelitis [10].

## Conclusions

Pneumorrhachis (PR) is a rare radiographic finding of air within the spinal canal. The location of PR is difficult to determine on computed tomography (CT) but has important clinical implications. The air within the subarachnoid space (intradural PR) is only seen with severe injuries and may present with neurological symptoms. The air within the epidural space (extradural PR) can be caused by a variety of pathologies and is usually asymptomatic. PR itself is usually self-limiting, as the air is spontaneously reabsorbed. Its discovery should warrant a thorough investigation of underlying injuries or disease processes, including emphysematous infections. Emphysematous cystitis (EC) is also a radiographic diagnosis whereby emphysematous changes are usually confined to the bladder wall and lumen. Severe EC infections may cause air to dissect into the surrounding soft tissues and, as seen here, into the epidural space of the spinal canal.
